# Testing the Functional Model of Bone Development: Direct and Mediating Role of Muscle Strength on Bone Properties in Growing Youth

**DOI:** 10.3390/ijerph18063154

**Published:** 2021-03-18

**Authors:** Izabella A. Ludwa, Kevin Mongeon, Malcolm Sanderson, Luis Gracia Marco, Panagiota Klentrou

**Affiliations:** 1 Centre for Bone and Muscle Health, Brock University, St. Catharines, ON L2S 3A1, Canada; iludwa@brocku.ca; 2 Faculty of Health Sciences, University of Ottawa, Ottawa, ON K1N 6N5, Canada; kevin.mongeon@uottawa.ca; 3 Department of Kinesiology, Brock University, St. Catharines, ON L2S 3A1, Canada; ms16ot@brocku.ca; 4 Department of Physical Education and Sports, PROFITH “PROmoting FITness and Health through Physical Activity” Research Group, Sport and Health University Research Institute (iMUDS), University of Granada, 18071 Granada, Spain; lgracia@ugr.es; 5 Instituto de Investigación Biosanitaria ibs.GRANADA, 18012 Granada, Spain

**Keywords:** children, bone development, muscle strength, radial speed of sound, tibial speed of sound, bone turnover

## Abstract

This study examines the functional model of bone development in peri-pubertal boys and girls. Specifically, we implemented a mixed-longitudinal design and hierarchical structural models to provide experimental evidence in support of the conceptual functional model of bone development, postulating that the primary mechanical stimulus of bone strength development is muscle force. To this end, we measured radial and tibial bone properties (speed of sound, SOS), isometric grip and knee extensors strength, bone resorption (urinary NTX concentration), body mass index (BMI), somatic maturity (years from peak height velocity) and skeletal maturity (bone age) in 180 children aged 8–16 years. Measurements were repeated 2–4 times over a period of 3 years. The multilevel structural equation modeling of 406 participant-session observations revealed similar results for radial and tibial SOS. Muscle strength (i.e., grip strength for the radial and knee extension for tibial model) and NTX have a significant direct effect on bone SOS (β = 0.29 and −0.18, respectively). Somatic maturity had a direct impact on muscle strength (β = 0.24) and both a direct and indirect effect on bone SOS (total effect, β = 0.30). Physical activity and BMI also had a significant direct impact on bone properties, (β = 0.06 and −0.18, respectively), and an additional significant indirect effect through muscle strength (β = 0.01 and 0.05, respectively) with small differences per bone site and sex. Muscle strength fully mediated the impact of bone age (β = 0.14) while there was no significant effect of energy intake on either muscle strength or bone SOS. In conclusion, our results support the functional model of bone development in that muscle strength and bone metabolism directly affect bone development while the contribution of maturity, physical activity, and other modulators such as BMI, on bone development is additionally modulated through their effect on muscle strength.

## 1. Introduction

The functional model of bone development postulates that the primary mechanical stimulus of bone strength development during growth comes from muscle [1,2]. According to this model, bone properties are regulated by a feedback loop between tissue strain (e.g., resulting from muscle contraction) and bone strength [2]. This suggests that the growth of bone and muscle are closely associated, and that bone adapts its strength in response to the muscle forces placed upon it [3,4]. Cross-sectional studies have demonstrated positive relationships between bone and muscle properties in youth [3,5,6,7]. Furthermore, a few longitudinal studies have suggested a temporal association between muscle and bone development [8,9]. Temporal association is the potential causal or contributory relationship between the onset of muscle and bone development, i.e., which comes first, eventually contributing to the other. To this end, the peak rate of increase in muscle mass, and presumably, muscle strength, has been shown to occur before the peak rate of increase in bone mass [9] and strength [10]. Indeed, although most of the longitudinal studies have relied on growth velocities to support the hypothesis of the temporal association between muscle and bone, the consensus is that the changes in muscle development precede changes in bone development [10,11,12], and that muscle strength influences bone strength. As previously suggested, the influence of muscle strength on bone may be due to higher density of enzymatic collagen cross-links in children compared with adults, which favors the elasticity of the collagen, and consequently, the bone’s resistance to mechanical loading [13]. However, a collagen matrix with more immature versus mature cross-links, as found in children, along with higher bone turnover, is more likely to deform before fracture, leading to an increased risk for plastic bending fractures of children’s cortical long bones [14]. It is, therefore, important to understand the role of collagen cross-links in the muscle-bone relationship during childhood and adolescence.

Studies that have examined the muscle-bone unit have used radiation-based technologies to indirectly measure bone strength using size-related measures (areal bone mineral density, content, area). However, the measurement of bone mineral density (BMD) and content (BMC) in children is problematic because it is influenced by the size of the bones, which varies according to the somatic maturity, i.e., the age from peak height velocity (PHV) of the child [15]. In addition, the bone mineral density is not correlated with the bone micro-architecture [15]. Transaxial quantitative ultrasound (QUS) measures the speed of sound (SOS) along the bone, making its assessment independent of bone size, which is important when comparing children of different ages [16,17]. QUS outcomes reflect both quantitative and qualitative properties, including density, elasticity, and microarchitecture of bone [16,17,18,19]. Specifically, the SOS measurements are related to BMD and internal structure [20], but not to cortical thickness [21]. QUS has been shown to be useful for screening of bone fragility in youth [22] and has previously been used to examine the effect of exercise and physical activity on various bones (e.g., tibia) and in different age groups, including youth [23,24,25,26,27,28]. In addition, studies examining the muscle-bone unit have also typically used measures of muscle size (e.g., muscle cross-sectional area or lean body mass), rather than muscle function as muscle strength typically scales with muscle size [29]. Muscle cross-sectional area (MCSA) and vertical jump test have, respectively, been used to examine the relationship between muscle size and muscle power with tibial bone strength in children [6], and adolescents [5]. These cross-sectional studies used peripheral quantitative computed tomography (pQCT) to provide quantitative measures of bone strength indices along the length of the tibia and demonstrated positive associations for both muscle size and power with lower extremity bone strength [5,6]. Along these lines, several cross-sectional studies conducted in healthy children have demonstrated positive associations between grip strength, as a measure of muscle strength at the forearm, and whole-body BMC [30,31], upper arm BMC [30], and radial SOS [32].

The use of mediation analysis in a longitudinal design is advantageous as it allows us to elucidate the various factors that directly and indirectly affect bone properties and muscle strength, which can expand our understanding of bone development. However, no longitudinal study has examined the relationship between muscle strength and both radial and tibia bone properties in children and adolescents, using mediation analysis. Only one study has used mediation analysis to examine the mediating effect of MCSA on the association of pQCT-derived tibial bone strength with muscle power in adolescent males and females, albeit this study was cross-sectional [5]. Therefore, the present study used mediation analysis in a mixed-longitudinal design, to examine the functional model of bone development in peri-pubertal boys and girls. Specifically, we developed hierarchical structural models to investigate the underlying relationships between muscle strength and non-mechanical factors that may modulate the effects on bone strength. The growing period is ideal to examine the underlying relationships between tissue strain and bone strength because physical growth forces the homeostatic system to continually adapt to external challenges [2].

## 2. Materials and Methods

### 2.1. Research Design and Participants

The study utilized a mixed-longitudinal design. Children and adolescents aged 8–16 years were recruited from schools located in Southern Ontario, Canada. Data were collected from participants annually at Brock University’s Applied Physiology Laboratory. To minimize potential seasonal effects, data collection took place during the spring and fall months [33]. The first data collection session took place in the spring of 2010 and last session in the spring of 2013. As a result, four data collection sessions occurred during the spring and three during the fall. Before participating in the study, all participants and their parent/guardian signed the informed consent and assent forms. The study and all its procedures were reviewed and approved by the Brock University Research Ethics Board.

Each data collection session involved two participant-visits, one week apart. During the first visit, anthropometric measures were taken, and isometric knee extensor strength was determined. Participants also completed a questionnaire regarding potential medical concerns and physical activity habits. Participants were also provided with a sterile collection cup for a urine sample. During the second visit, urine samples were collected, and grip strength tests, bone ultrasound scans, and a 24-hour recall nutritional interview were conducted.

Ninety-four children or adolescents participated in the spring sessions and 86 participated in the fall sessions, resulting in 180 total participants (92 boys, 88 girls). Of the 180 participants, 36 attended one session, 53 attended two, 72 attended three, and 19 attended four sessions, resulting in 434 participant-session observations. However, not all participant-sessions resulted in data suitable for analysis purposes. Six observations were omitted because the participant was identified to have Type 1 Diabetes, eight observations were omitted because the participant suffered a fracture, eleven observations were omitted because the SOS value could not be detected at the measurement site, and three observations were omitted because the participant missed the second visit and did not have all the SOS values. Grip-strength measurements were not taken during the Fall 2010 season. Therefore, 43 observations do not include a grip-strength measurement. Urinary levels of bone resorption were unavailable for 29 observations for which the participant did not provide a sample. In the end, the data available for analysis consisted of 406 participant-session observations with radial and tibial SOS, of which 306 observations include grip-strength measurements, and 376 include urinary concentration of cross-linked N-telopeptides of bone type I collagen (NTX).

### 2.2. Measurements

#### 2.2.1. Bone Properties

Transaxial quantitative ultrasound (QUS, Sunlight Omnisense™ 7000S, Sunlight Medical, Tel Aviv, Israel) was used to assess bone SOS (m/s) along the bone at the distal 1/3 of the radius and at the mid-tibia of the dominant limbs, as previously described [34]. The strength of bone was determined by the shortest time elapsed between the transmission and reception of the signal transmitted, with faster transmissions reflecting stronger bone [21]. Wide scans of 140 degrees were performed around the radius at the midpoint between the olecranon process and the tip of the third phalanx. To measure the SOS of the tibial shaft, a line was marked midway between the apex of the top of the knee and the sole of the foot, with the subject in a sitting position and the knee at a 90° angle. The probe was placed parallel to the tibial bone surface, and a scan from the medial to lateral side was performed. All measurements consisted of at least three consistent cycles. A system quality verification of the QUS was performed with a Perspex phantom before the first test of each day. Although every effort was made for the same researcher to perform all QUS measurements for the duration of the longitudinal study, this was not always possible. Thus, one researcher performed almost all SOS measurements with an intra-operator coefficient of variation in 10 children of 2% and an interclass correlation coefficient of 0.98. The inter-operator coefficient of variation was 3%.

#### 2.2.2. Muscle Strength

Maximal dominant forearm strength was assessed by a hand-held dynamometer to determine maximal isometric grip force. The device handle was adjusted to the participant’s grip size. The test was performed with the participant in a standing position with their dominant arm abducted at about 45 degrees with their elbow extended [35]. Participants were instructed to squeeze the instrument as hard as possible for 3 s. Measurements were recorded to the nearest 0.5 kg. Contractions were performed 3 times and the best attempt was recorded as the absolute maximal isometric grip force. Proper technique was monitored to minimize postural compensations and were corrected as necessary. Isometric grip strength has been widely used in pediatric studies [36], with a high test-retest reliability reported in untrained children [37], and untrained and trained adolescents [37,38].

Isometric knee extensors strength measurements were performed on the dominant leg, using a Biodex System III dynamometer (Biodex, Shirley, NY, USA). The participants were seated in the dynamometer’s chair and stabilized using a cross-hip strap and two diagonal, cross-chest shoulder straps. The dynamometer lever-arm contact pad was adjusted to ~3 cm above the lateral malleolus via an ankle strap. The lever’s axis of rotation was aligned with the knee’s axis (femur’s lateral condyle). The knee was then set at a 90° starting position (180° = full extension). A familiarization and warm-up protocol consisted of several submaximal isometric contractions and two maximal isometric contractions. If a participant did not feel comfortable with the movement or the protocol or exhibited performance inconsistency by the end of the familiarization sets, additional trials were administered. The subsequent testing consisted of eight 3 s maximal isometric knee extensions at 90°, separated by a minimum of 30 s rest between repetitions. Prior to each contraction, participants were instructed to “kick out as fast and then as hard as possible” from a completely relaxed state. Verbal encouragement was given, along with visual torque-level feedback on the Biodex monitor. Torque signals were recorded prior to and throughout each contraction. The highest peak torque was recorded.

#### 2.2.3. Bone Resorption

We measured urinary concentration of cross-linked N-telopeptides of bone type I collagen (NTX) to monitor bone resorption, i.e., osteoclast activity. NTX was measured in first morning mid-stream urine samples and analyzed in duplicate using enzyme-linked immunosorbent assay (ELISA) kits (Osteomark^®^ Ntx Urine Assay, Alere Scarborough, Inc., Scarborough, ME, USA). All assayed plates were read using the same microplate reader and absorbencies were analyzed using GraphPad Prism (GraphPad Software, Inc., La Jolla, CA, USA). Urinary creatinine was analyzed in duplicate using a creatinine colormetric assay kit (MicroVue™, Quidel Corporation, San Diego, CA, USA) based on a modified Jaffe method. NTX values were corrected for urinary creatinine with results reported as nmol bone collagen equivalents (BCE)/mmol creatinine. The intra-assay and inter-assay coefficient of variation for NTX was, 2.5% and 11.6%, respectively.

#### 2.2.4. Anthropometry and Maturity

All anthropometric measurements were performed by the same investigator. Body mass was measured to the nearest 0.1 kg using a calibrated balance beam scale (Zenith Digital Scale). Standing and seated height were measured using a stationary stadiometer (Ellard Instrumentation, Monroe, WA, USA) and recorded to the nearest 0.1 cm. Somatic maturity was then determined from the maturity offset (years from age of PHV), which was estimated using sex-specific regression equations [39]. It was assessed from measurements of height, seated height, leg length, body mass and chronological age, and it was adjusted in accordance with the measurement at the age closest to the estimated age of PHV.

Bone age (in years) was determined using the Sunlight BonAge Ultrasound System (Tel Aviv, Israel), based on the process of ossification at the radial and ulnar epiphyses during growth, as previously described [40]. Briefly, the non-dominant wrist was aligned between two ultrasound transducers, at the level of the ulnar styloid process, as determined by a technician. Speed of ultrasound was measured across the radial and ulnar epiphyses over several (7–11, depending on bone size) cycles and bone age was computed based on a proprietary sex- and ethnicity-based algorithm to provide a numeric bone age result in years and months. A system calibration procedure was performed prior to testing each subject. This technique has been shown to produce an accurate assessment of skeletal maturity, compared with traditional radiographic methods [41].

#### 2.2.5. Nutrition and Physical Activity

Dietary intake was evaluated using a 24-h recall interview as previously described [36]. In brief, participants were asked to recall everything consumed (including foods, beverages, sauces, and condiments) the previous day from morning to bedtime. Prior to answering the 24-h dietary recall, participants were asked if the last 24 h were typical for their diet. If it were not a typical day (e.g., birthday party, family gathering, eating out), they reported two days prior to the recall day. Pictures representing different portion sizes of foods, sizes and measurements of various kitchenware models were used to ascertain the most accurate amount of food that was consumed. Dietary analysis was conducted by the same investigator using the Nutritionist Pro^TM^ software (Axxya Systems, Redmond, WA USA) to estimate total daily energy intake (kcal), as well as daily protein (g), calcium (mg) and vitamin D (µg) intake.

Habitual physical activity was self-reported using the Physical Activity Questionnaire for Children (PAQ-C). This is a brief, 7-day recall instrument that was developed to assess general levels of moderate to vigorous physical activity during the school year for students in grades 4 to 8 and children approximately 8 to 14 years of age [42]. PAQ-C has demonstrated adequate validity and internal consistency and is recommended for use in longitudinal large-scale research [42,43]. It provides a summary physical activity score derived from nine items, each scored on a 5-point scale, but does not provide an estimate of caloric expenditure or specific frequency, time, and intensity information. The Godin-Shephard Leisure Time Exercise Questionnaire [44] was also used to assess weekly physical activity energy expenditure. Participants were asked to indicate the number of times in a typical week they engaged in mild, moderate and strenuous physical activity for at least 15 min. These frequencies were then multiplied by estimated energy consumption values (in metabolic equivalents [MET]) and summed to obtain total weekly leisure time physical activity metabolic equivalent (WAeq) scores. This questionnaire has demonstrated adequate validity and reliability in children and adults [45,46,47]. The two measures of physical activity were necessary because physical activity impacts both muscle strength and bone development, so the PAQ-C score was used in the bone models and WAeq score was used in the muscle models.

### 2.3. Statistical Analysis

The repeated sampling of participants resulted in a hierarchical data set that consisted of within (level 1) and between (level 2) measurement variations. Table 1 outlines the variables used in the analysis based on the functional model of bone development, their means, and their standard deviations within and between participants. Shapiro-Wilk’s tests did not reject the null hypothesis that the radial and tibial bone data are normally distributed.

### 2.4. Empirical Model

**Figure 1:**
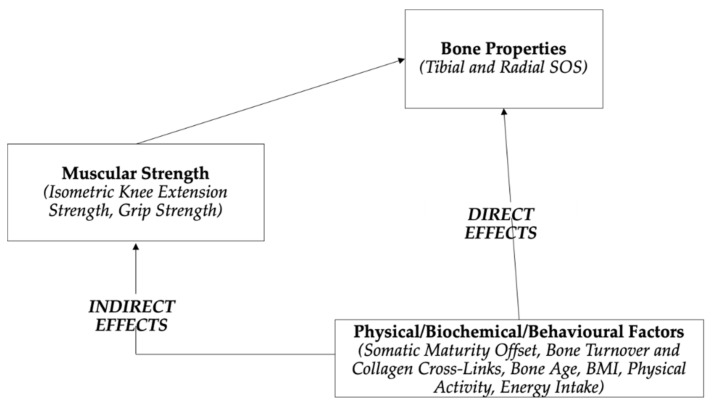
Modified functional model of bone development in children and adolescents. Items in italics are variables used to reflect each factor.

The functional model of bone development provides the conceptual framework to model changes in bone properties, as reflected by radial and tibial SOS. Rather than a direct causal effect between modulators and bone properties, the functional model of bone development postulates that modulators (i.e., physical/behavioural factors) influence muscle strength (grip strength for the radial and knee extension for tibial model) and bone turnover, which in turn influence bone properties. The functional model of bone development considers bone turnover as part of the regulatory feedback loop influencing bone properties [2]. Our empirical model has modified the functional model of bone development (Figure 1) to incorporate bone turnover and collagen cross-links, as one of the potential modulators influencing bone properties.

A multilevel structural equation model (SEM) was developed to test the intrinsic relationships between modulators, muscle strength, and bone properties. In brief, SEM is a multivariate statistical technique used to estimate a system of equations and test hypotheses about the relationships among variables. To do so, SEM explicates the direct relationships between observed variables and the covariance relationships between unobserved (latent) variables. Models were constructed using Stata 14′s Generalized Structural Equation package (see [48,49] for an overview of structural equation modeling). The mediated models attempt to disentangle average effects on bone properties into effects that directly impact bone properties and effects that indirectly arise through muscle strength to impact bone properties. The general SEM to describe changes in bone properties can be expressed as

(1) $$Y_{i,t}=\beta_{0}\iota +M_{i,t}’\beta_{1}+\Theta Z_{i,t}\iota +\gamma_{i}\iota +\epsilon_{i,t}$$

(2) $$M_{i,t}=W_{i,t}’\Omega \iota \;+\delta_{i}\iota +\mu_{i,t}$$

The $Y_{i,t}$ denotes ith participant’s bone properties measurement on the tth occasion, and the $M_{i,t}$ term donotes his or her muscle strength measurement. The $Z_{i,t}$ and $W_{i,t}$ terms are matricies of modulators and control variables that potentially influence bone properties or muscle strength changes, respectively. The β and Ω terms denote the unknown fixed parameters, the $\epsilon_{i,t}$ and $\mu_{i,t}$ terms denote the unobserved within-participant (level 1) residuals, and the $\gamma_{i}$ term denotes the unobserved random participant-effects (level 2). The random participant-effects are assumed to be normally distributed with a mean of zero and independent of the covariates.

In the case of two bone and muscle strength measurements, the model can be expressed as $Y_{i,t}=\left[\begin{array}{c} y_{1}{}_{i,t}\\ y_{2}{}_{i,t} \end{array}\right]$, where $y_{1}{}_{i,t}$ and $y_{2}{}_{i,t}$ denote radial SOS and tibial SOS measurements, and $M_{i,t}=\left[\begin{array}{c} m_{1}{}_{i,t}\\ m_{2}{}_{i,t} \end{array}\right]$, where $m_{1}{}_{i,t}$ and $m_{2}{}_{i,t}$ denote the isometric grip strength and knee extensor measurements. In the two dimensional case $\epsilon_{i,t}=\left[\begin{array}{c} \epsilon_{1}{}_{i,t}\\ \epsilon_{2}{}_{i,t} \end{array}\right]$, $\mu_{i,t}=\left[\begin{array}{c} \mu_{1}{}_{i,t}\\ \mu_{2}{}_{i,t} \end{array}\right]$, $\Sigma =\left[\begin{array}{cc} \sigma_{\epsilon_{1}}^{2} & \sigma_{\epsilon_{1},\epsilon_{2}}\\ \sigma_{\epsilon_{2},\epsilon_{1}} & \sigma_{\epsilon_{2}}^{2} \end{array}\right]$, and $\Psi =\left[\begin{array}{cc} \sigma_{\mu_{1}}^{2} & \sigma_{\mu_{1},\mu_{2}}\\ \sigma_{\mu_{2},\mu_{1}} & \sigma_{\mu_{2}}^{2} \end{array}\right]$, where Σ denotes the variance-covariance matrix between the bone properties residuals and Ψ denotes the variance-covariance matrix between the muscle strength residuals. The $\iota_{i,t}=\left[\begin{array}{c} 1\\ 1 \end{array}\right]$ term ensures the matricies conform.

As previously noted, PAQ-C score accounts for physical activity in the bone models and WAeq score accounts for physical activity in the muscle model. We could not have the same variable in both models because the variables are significant in their respective equations, in that physical activity impacts both muscle strength and bone development. 

## 3. Results

The empirical results are presented in Table 2, Table 3 and Table 4. Each table presents the estimation results from the bone and muscle strength equations (i.e., Equations (1) and (2)), as well as the computed indirect and total effects. To facilitate straightforward comparison of the causal effects, all variables were transformed/standardized to a mean of zero and standard deviation of one (z-score). Conclusions based on non-standardized values are in line with the standardized values analysis.

The overall results (Table 2) are consistent with the relationships postulated by the functional model of bone development model, in that modulators contribute to the development of muscle strength, which in turn, contribute to bone development. Specifically, muscle strength (i.e., grip strength for the radial and knee extension for tibial model) and NTX directly influenced bone SOS changes, with muscle strength having the largest positive effect on bone properties and NTX having a negative direct effect on bone properties (Table 2). Physical activity also had a direct impact on bone properties, as indicated by PAQ-C score (β = 0.056), and an indirect effect through muscle strength, as indicated by the WAeq score (β = 0.013). Muscle strength partially mediated the effect of somatic maturation on bone SOS changes. Somatic maturation was directly associated with both muscle strength and bone SOS while it also had an indirect effect on bone properties, for a total effect of β = 0.302. In addition, muscle strength fully mediated the impact of bone age on bone properties (β = 0.138). BMI was associated with increased muscle strength and decreased bone SOS, holding other factors constant. However, its indirect impact through muscle strength was positive (β = 0.05). In addition, muscle strength fully mediated the impact of energy intake on bone properties, although the effect was not significant (Table 2).

The bone properties analysis results for boys and girls, separately, are presented in Table 3. Excluding BMI, a Chow test did not reject the null hypothesis that the boy and girl bone property coefficients are equal, suggesting that inferences based on the total cohort are valid across sexes. Although broad inferences concerning the relationships postulated by the functional model of bone development can be generalized across sexes, some notable differences exist between boys and girls. BMI impacts bone properties development in boys significantly more than in girls (*p* = 0.02). Somatic maturity impacts muscle strength development in boys more than in girls (Table 3). However, this difference in the total effect on bone properties was not significant (β = 0.13[0.44–0.31], *p* = 0.19). In contrast, the order of magnitude of the indirect effect of physical activity (WAeq) and of skeletal maturity (i.e., bone age) on bone SOS is greater in girls than in boys (Table 3).

Test results are also similar across radial and tibial SOS (Table 4). In terms of the impact of muscle strength on bone properties, both the grip strength effect on radial properties and the knee extensor effect on tibial properties were significant and of similar magnitude. The impact of bone resorption (i.e., urinary NTX) and somatic maturation were not significantly different between the radial and tibial equations. Somatic maturation had a greater influence on knee extensor strength than on grip strength, causing the total effect of somatic maturation on the tibial SOS to be greater than on the radial SOS. The PAQ-C score had a significant effect on radial SOS, but not on the tibial SOS, and the same is true for the effect of WAeq on muscle strength. At the radius, BMI had no significant direct or total effect on SOS, although its indirect effect was significant (Table 4). At the tibia, BMI had a significant direct and indirect impact on SOS. PAQ-C score (direct impact of physical activity) was found to be significant at the radius, but not at the tibia. The significant indirect effect of physical activity (WAeq), albeit small, was also only present at the radius. The indirect effect of bone age remained significantly mediated for both radial and tibial SOS. Energy intake had a significant direct effect only on knee extension (Table 4).

## 4. Discussion

**Figure 2:**
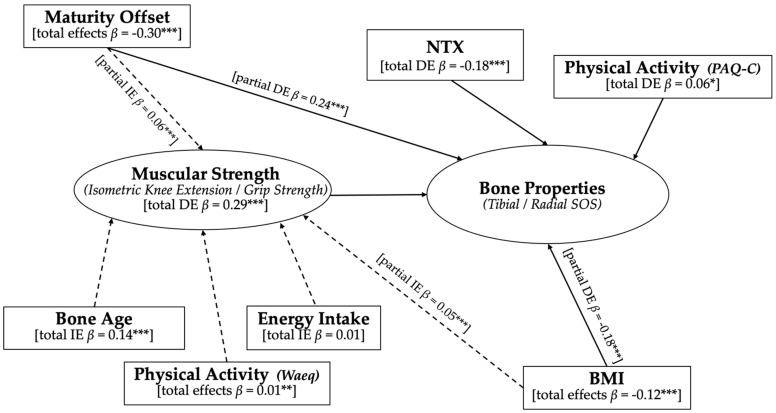
Diagram representing empirical results supporting the modified functional model of bone development in children and adolescents (total cohort). Solid arrows show direct effects (DE) of modulators on bone properties. Dashed arrows represent the indirect effects (IE) of modulators on bone properties that are either fully or partially mediated by muscle strength. * denotes *p* < 0.05; ** denotes *p* < 0.01; *** denotes *p* < 0.001. NTX = cross-linked N-telopeptides of bone type I collagen; PAQ-C = physical activity questionnaire for children; PHV = peak height velocity; BMI = body mass index; WAeq = weekly leisure time physical activity metabolic equivalent.

To our knowledge, this is the first study to longitudinally investigate the relationship between muscle strength and non-mechanical modulators with tibial and radial bone properties in children and adolescents. A unique aspect of the study was the use of our empirical model which allowed us to examine the indirect and direct effects of multiple variables. Overall, our results support the functional model of bone development. Specifically, this empirical model was used to determine the direct effect of muscle strength on bone properties and helped to tease out how much of the effect of the modulating variables is mediated by muscle strength (Figure 2). Muscle strength and collagen cross-links (NTX) had significant direct effects, positive and negative, respectively, on bone properties. Somatic maturity and BMI were partially mediated by muscle strength but had significant direct and indirect effects on bone properties, which differed by sex and bone site. This was also the case when considering the impacts of the different measures of physical activity (PAQ-C, WAeq) on bone properties. Bone age and energy intake effects were found to be fully mediated by muscle strength, although the effect of energy intake on bone properties was not significant.

A benefit of the empirical model is that it helped to separate the influence of impact forces (like physical activity) from the direct effect of muscle strength. Indeed, the direct effects of muscle strength were greater than the combined direct (PAQ-C) and indirect (WAeq) effects of physical activity on bone properties (Figure 2). These direct effects support the notion of muscle forces placing some of the largest physiological loads on bone, causing bone to adapt and increase in strength [3,4]. Physical activity was found to have significant direct (PAQ-C) and indirect effects (WAeq) at the radius only, and in girls. The significant effect on radial SOS, as opposed to the weight-bearing bone of the tibia, and in girls, may be a result of progressive decreases in physical activity through childhood and adolescence that is more profound, and probably more consistent, among females than males [34,50].

Interestingly, somatic maturity impacts muscle strength in boys more than in girls, however its effect on bone properties was similar between sexes. Overall, somatic maturity had the greatest total effect on bone properties, showing both direct and indirect effects, mediated by muscle strength (Figure 2). Our previous cross-sectional analysis, in this same group of children, also showed that maturity offset was the greatest predictor (explaining 12% of the variance) of radial SOS [32]. However, in the present longitudinal analysis, it was grip strength that had the greatest significant impact on radial SOS compared with somatic maturity and other modulators. Conversely, at the tibia, somatic maturity was found to have the strongest impact on SOS. Specifically, the direct impact of somatic maturity and knee extension strength were similar, but when the indirect effects were considered, total somatic maturity effects were greater than knee extension strength alone (β = 0.38 vs. 0.30, respectively). On the other hand, the finding that the significant effect of bone age on bone properties was fully mediated by muscle strength was surprising. As bone age reflects skeletal maturity, direct effects on bone properties would have been expected. According to the mechanostat model, bone development is driven by increases in both bone length and muscle force [1,2]. Although we did not measure bone length specifically, the ultrasonic assessment of bone age was based on the process of ossification at the radial and ulnar epiphyses [40]. Together these findings suggest the growth of bone and muscle are closely associated.

NTX consistently had a significant negative direct effect on bone properties. This negative relationship may be indicative of typical maturational changes, whereby NTX concentrations decrease with age as bone strength increases. Although we did not measure the quantity of immature versus mature cross-links, higher density of enzymatic N-terminal cross-links of collagen is suggested to maximize bone’s mechanical properties (elasticity and plasticity) to account for a larger risk of falls or green stick fractures in children [13,14]. Interestingly, the direct effect of NTX was observed to be greater at the radius than tibia (β = −0.25 vs. −0.15, respectively). This longitudinal result is consistent with the previously reported cross-sectional findings [32] from our lab that found NTX, in addition to grip strength, to be a key predictor of radial SOS. With age, the conversion from immature to mature cross-links would result in lower overall NTX levels, making bone stronger to allow for more intense physical activities [14]. However, the remodeling rate of bone is also dependent on the mechanical action on osteocytes that leads to the activation of osteoclasts [14], making it difficult for us to determine if the effects of NTX is the result of an elevated maturational turnover or the impact of grip strength on radial SOS.

In the present longitudinal study, BMI had a direct negative effect on the development of bone properties, especially at the tibia and significantly more in boys than in girls. This contradicts Ivuskans et al. [51], who reported positive moderate to large correlations between BMI and whole-body BMD and BMC in normal weight and overweight peri pubertal boys. It is unclear why our group of boys demonstrated significant negative direct and total effects of BMI. It is possible that this finding reflects the nature of BMI as a measure of body size that cannot distinguish the effect of fat mass deposition from muscle mass development. This assumption is supported by a closer examination of our results, which reveals positive indirect effects of BMI on both radial and tibial SOS in both sexes, suggesting that any positive effect of BMI on bone properties may be applied through increases in muscle strength due to muscle mass development. Future studies should include measures of body size and composition to distinguish the contribution of each of the tenants in the functional model of bone development in children. Finally, adequate energy and dietary intakes of protein, calcium and vitamin D are important for both muscle and bone development [52,53]. However, in the current study, only the direct effect of energy intake on knee extension strength was found to be significant, which was not surprising given our participants were healthy, typically developing children without signs of protein and energy deficits.

A limitation to this study is that markers of bone formation were not measured, which in addition to our analyzed marker of bone resorption (NTX), could have provided a more accurate reflection of bone turnover as part of the regulatory feedback loop in the functional model of bone development. Using accelerometry, in addition to our questionnaires, would have helped to elucidate the observed different effects of physical activity on radial and tibial SOS by including an objective measurement of predominantly weight-bearing activity and ground reaction forces. The retrospective recall of nutrition is also a limiting method, which can be particularly problematic for our younger participants. The advantage of using a hierarchical model in this longitudinal design is that there are many participants with multiple time points, and by imposing the partially mediated structural equation model we can elucidate the various factors that directly and indirectly affect bone properties and muscle strength and expand our understanding of bone development.

## 5. Conclusions

This study implemented a mixed-longitudinal design, and hierarchical structural models to confirm the direct and mediating role of muscle strength on bone properties in growing children and adolescents, in support of the conceptual functional model of bone development. Our results demonstrated these effects varied by sex and bone site. In both males and females, the direct effects of muscle strength were similar to that of maturity, except for the radius. Indeed, grip strength had the greatest impact on radial SOS compared with somatic maturity and other modulators. Somatic maturity showed the greatest total (direct and indirect) effects on bone properties, specifically at the tibia. In contrast, the impact of skeletal maturity (i.e., bone age) on bone SOS was fully mediated by muscle strength. NTX had a significant negative direct effect on bone SOS that appeared greater at the radius. Physical activity was found to affect bone properties significantly at the radius, and in girls, whereas BMI, as a surrogate measure of body size, played a larger role on the tibia, and in boys. Surprisingly, there was no significant effect of energy intake on either muscle strength or bone SOS. These findings add to our global understanding of the different factors effecting bone development in youth using non-radiating and non-invasive ultrasound techniques that may enhance diagnosis in pediatrics [15]. The strong presence of NTX in our results demonstrate that enzymatic collagen cross-links could be enhanced using mechanical loading such as musculoskeletal rehabilitation [13]. Our results show a greater sensitivity of bone properties towards grip strength, advocating for potential interventions to further improve radial bone properties. Finally, evaluating bone health from the perspective of a functional muscle-bone unit may increase the sensitivity of fracture prediction in this population.

## Figures and Tables

**Table 1:** Summary statistics (means and standard deviations) of all variables used in the analysis, within and between participants.

Variable	Mean	SD	SD within	SD between
Age (years)	11.8	2.0	1.0	1.5
Height (cm)	152.1	13.6	7.4	10.5
Weight (kg)	46.2	14.4	8.2	11.7
Maturity offset (years from age of PHV)	−0.9	2.0	0.9	1.9
Radial SOS (m/s)	3816.1	100.0	51.4	86.0
Tibial SOS (m/s)	3687.4	109.4	49.8	96.7
Grip strength (kg)	23.4	7.40	3.2	7.0
Knee extension (kg)	131.5	57.7	22.3	56.6
NTX (nmol BCE/mmol creatinine)	539.8	263.6	169.9	213.7
Energy intake (kcal/day)	1593.4	479.5	297.6	370.0
WAeq (MET)	73.8	39.6	24.8	32.9
PAQ-C (score)	0.1	0.3	0.2	0.2
BMI (kg/m^2^)	19.5	3.7	1.2	3.6
Bone age (years)	12.0	2.3	1.1	2.2

SOS = speed of sound; NTX = cross-linked N-telopeptides of bone type I collagen; BCE = bone collagen equivalents; PHV = peak height velocity; WAeq = weekly leisure time physical activity metabolic equivalent; PAQ-C = physical activity questionnaire for children; BMI = body mass index.

**Table 2:** Functional model of bone development results (total cohort). Values are β-coefficients with standard errors in parentheses.

Modulators	Bone Properties	Muscle Strength	Indirect Effects on Bone SOS	Total Effects on Bone SOS
Muscle strength (kg)	0.288 *** (0.061)			
NTX (nmol BCE/mmol creatinine)	−0.184 *** (0.033)			
PAQ-C (score)	0.056 * (0.029)			
Maturity offset (years from aPHV)	0.241 *** (0.052)	0.213 *** (0.037)	0.061 *** (0.017)	0.302 *** (0.046)
BMI (kg/m^2^)	−0.178 *** (0.056)	0.188 *** (0.033)	0.054 *** (0.014)	−0.124 ** (0.054)
Bone age (years)		0.480 *** (0.038)	0.138 *** (0.031)	0.138 *** (0.031)
WAeq (MET)		0.047 ** (0.021)	0.013 ** (0.007)	0.013 ** (0.007)
Energy intake (kcal/day)		0.032 (0.023)	0.009 (0.007)	0.009 (0.007)

* denotes *p* < 0.05; ** denotes *p* < 0.01; *** denotes *p* < 0.001; SOS = speed of sound; NTX = cross-linked N-telopeptides of bone type I collagen; BCE = bone collagen equivalents; PAQ-C = physical activity questionnaire for children; aPHV = age from peak height velocity; BMI = body mass index; WAeq = weekly leisure time physical activity metabolic equivalent.

**Table 3:** Functional model of bone development results for boys and girls, separately. Values are β-coefficients with standard errors in parentheses.

Modulators	Boys (N = 92)				Girls (N = 88)			
	Bone Properties	Muscle Strength	Indirect Effect on Bone SOS	Total Effect on Bone SOS	Bone Properties	Muscle Strength	Indirect Effect on Bone SOS	Total Effect on Bone SOS
Muscle strength (kg)	0.250 *** (0.093)				0.288 *** (0.095)			
NTX (nmol BCE/ mmol creatinine)	−0.185 *** (0.045)				−0.163 *** (0.048)			
PAQ-C (score)	0.052 (0.035)				0.044 (0.047)			
Maturity offset (years from age of PHV)	0.264 ** (0.110)	0.709 *** (0.073)	0.177 *** (0.067)	0.441 *** (0.073)	0.263 *** (0.071)	0.169 *** (0.044)	0.049 ** (0.021)	0.311 *** (0.066)
BMI (kg/m^2^)	−0.315 *** (0.077)	0.210 *** (0.046)	0.052 ** (0.023)	−0.263 *** (0.078)	−0.055 (0.082)	0.180 *** (0.039)	0.052 *** (0.020)	−0.003 (0.080)
Bone age (years)		0.192 *** (0.060)	0.048 ** (0.024)	0.048 ** (0.024)		0.425 *** (0.048)	0.122 *** (0.043)	0.122 *** (0.043)
WAeq (MET)		0.007 (0.022)	0.002 (0.006)	0.002 (0.006)		0.076 ** (0.033)	0.022 * (0.012)	0.022 * (0.012)
Energy intake (kcal/day)		0.007 (0.026)	0.002 (0.007)	0.002 (0.007)		−0.002 (0.030)	−0.0005 (0.0087)	−0.0005 (0.0087)

* denotes *p* < 0.05; ** denotes *p* < 0.01; *** denotes *p* < 0.001; SOS = speed of sound; NTX = cross-linked N-telopeptides of bone type I collagen; BCE = bone collagen equivalents; PAQ-C = physical activity questionnaire for children; PHV = peak height velocity; BMI = body mass index; WAeq = weekly leisure time physical activity metabolic equivalent.

**Table 4:** Functional model of bone development results for the radius and tibia separately (total cohort). Values are β coefficients with standard errors in parentheses.

	Radial				Tibial			
Modulators	SOS	Grip Strength	Indirect Effect on Bone SOS	Total Effect on Bone SOS	SOS	Knee Extension	Indirect Effect on Bone SOS	Total Effect on Bone SOS
Grip strength (kg)	0.257 *** (0.072)							
Knee extension (kg)					0.295 *** (0.071)			
NTX (nmol BCE/ mmol creatinine)	−0.251 *** (0.050)				−0.153 *** (0.038)			
PAQ-C (score)	0.092 ** (0.043)				0.033 (0.035)			
Maturity offset (years from age of PHV)	0.165 *** (0.063)	0.135 *** (0.046)	0.035 ** (0.015)	0.199 *** (0.059)	0.300 *** (0.059)	0.268 *** (0.041)	0.079 *** (0.023)	0.379 *** (0.052)
BMI (kg/m^2^)	−0.060 (0.067)	0.168 *** (0.039)	0.043 *** (0.015)	−0.017 (0.065)	−0.253 *** (0.061)	0.205 *** (0.036)	0.060 *** (0.017)	−0.192 *** (0.059)
Bone age (years)		0.563 *** (0.047)	0.145 *** (0.042)	0.145 *** (0.042)		0.426 *** (0.043)	0.126 *** (0.033)	0.126 *** (0.033)
WAeq (MET)		0.087 *** (0.029)	0.022 ** (0.010)	0.022 ** (0.010)		0.022 (0.024)	0.006 (0.007)	0.006 (0.007)
Energy intake (kcal/day)		0.012 (0.030)	0.003 (0.008)	0.003 (0.008)		0.045 * (0.026)	0.013 (0.008)	0.013 (0.008)

* denotes *p* < 0.05; ** denotes *p* < 0.01; *** denotes *p* < 0.001; SOS = speed of sound; NTX = cross-linked N-telopeptides of bone type I collagen; BCE = bone collagen equivalents; PAQ-C = physical activity questionnaire for children; PHV = peak height velocity; BMI = body mass index; WAeq = weekly leisure time physical activity metabolic equivalent.

## Data Availability

The data presented in this study are available on request from the corresponding author [PK] for researchers who meet the criteria for access to confidential data. The data are not publicly available due to REB restrictions.

## References

[1] Frost H.M. (1987). Bone “Mass” and the “Mechanostat”: A Proposal. Anat. Rec..

[2] Rauch F., Schoenau E. (2001). The Developing Bone: Slave or Master of Its Cells and Molecules?. Pediatr. Res..

[3] Schoenau E., Frost H.M. (2002). The “Muscle-Bone Unit” in Children and Adolescents. Calcif. Tissue. Int..

[4] Schoenau E., Fricke O. (2008). Mechanical Influences on Bone Development in Children. Euro. J. Endocrinol..

[5] Janz K.F., Letuchy E.M., Burns T.L., Francis S.L., Levy S.M. (2015). Muscle Power Predicts Adolescent Bone Strength: Iowa Bone Development Study. Med. Sci. Sports Exerc..

[6] Macdonald H., Kontulainen S., Petit M., Janssen P., McKay H. (2006). Bone Strength and Its Determinants in Pre- and Early Pubertal Boys and Girls. Bone.

[7] Schoenau E., Neu C.M., Mokov E., Wassmer G., Manz F. (2000). Influence of Puberty on Muscle Area and Cortical Bone Area of the Forearm in Boys and Girls. J. Clin. Endocrinol. Metab..

[8] Takei S., Taketomi S., Tanaka S., Torii S. (2020). Growth Pattern of Lumbar Bone Mineral Content and Trunk Muscles in Adolescent Male Soccer Players. J. Bone. Miner. Metab..

[9] Rauch F., Bailey D.A., Baxter-Jones A., Mirwald R., Faulkner R. (2004). The ‘Muscle-Bone Unit’ during the Pubertal Growth Spurt. Bone.

[10] Jackowski S.A., Faulkner R.A., Farthing J.P., Kontulainen S.A., Beck T.J., Baxter-Jones A.D.G. (2009). Peak Lean Tissue Mass Accrual Precedes Changes in Bone Strength Indices at the Proximal Femur during the Pubertal Growth Spurt. Bone.

[11] Xu L., Nicholson P., Wang Q., Alén M., Cheng S. (2009). Bone and Muscle Development During Puberty in Girls: A Seven-Year Longitudinal Study. J. Bone Miner. Res..

[12] Wang Q., Alén M., Nicholson P., Suominen H., Koistinen A., Kröger H., Cheng S. (2007). Weight-Bearing, Muscle Loading and Bone Mineral Accrual in Pubertal Girls—A 2-Year Longitudinal Study. Bone.

[13] Depalle B., Duarte A.G., Fiedler I.A.K., Pujo-Menjouet L., Buehler M.J., Berteau J.-P. (2018). The Different Distribution of Enzymatic Collagen Cross-Links Found in Adult and Children Bone Result in Different Mechanical Behavior of Collagen. Bone.

[14] Berteau J.-P., Gineyts E., Pithioux M., Baron C., Boivin G., Lasaygues P., Chabrand P., Follet H. (2015). Ratio between Mature and Immature Enzymatic Cross-Links Correlates with Post-Yield Cortical Bone Behavior: An Insight into Greenstick Fractures of the Child Fibula. Bone.

[15] Berteau J.-P., Baron C., Pithioux M., Launay F., Chabrand P., Lasaygues P. (2014). In Vitro Ultrasonic and Mechanic Characterization of the Modulus of Elasticity of Children Cortical Bone. Ultrasonics.

[16] Baroncelli G.I. (2008). Quantitative Ultrasound Methods to Assess Bone Mineral Status in Children: Technical Characteristics, Performance, and Clinical Application. Pediatr. Res..

[17] Foldes A.J., Rimon A., Keinan D.D., Popovtzer M.M. (1995). Quantitative Ultrasound of the Tibia: A Novel Approach for Assessment of Bone Status. Bone.

[18] Jaworski M., Lebiedowski M., Lorenc R.S., Trempe J. (1995). Ultrasound Bone Measurement in Pediatric Subjects. Calcif. Tissue Int..

[19] Prins S., Jørgensen H., Jørgensen L., Hassager C. (1998). The Role of Quantitative Ultrasound in the Assessment of Bone: A Review: Role of Quantitative Ultrasound in Assessment of Bone. Clin. Physiol..

[20] Gluer C.C., Wu C.Y., Jergas M., Goldstein S.A., Genant H.K. (1994). Three Quantitative Ultrasound Parameters Reflect Bone Structure. Calcif. Tissue Int..

[21] Njeh C.F., Hans D., Wu C., Kantorovich E., Sister M., Fuerst T., Genant H.K. (1999). An in Vitro Investigation of the Dependence on Sample Thickness of the Speed of Sound along the Specimen. Med. Eng. Phys..

[22] Rebocho L.M., Cardadeiro G., Zymbal V., Gonçalves E.M., Sardinha L.B., Baptista F. (2014). Measurement Properties of Radial and Tibial Speed of Sound for Screening Bone Fragility in 10- to 12-Year-Old Boys and Girls. J. Clin. Densitom..

[23] Daly R.M., Rich P.A., Klein R. (1997). Influence of High Impact Loading on Ultrasound Bone Measurements in Children: A Cross-Sectional Report. Calcif. Tissue. Int..

[24] Falk B., Galili Y., Zigel L., Constantini N., Eliakim A. (2007). A Cumulative Effect of Physical Training on Bone Strength in Males. Int. J. Sports Med..

[25] Holmes B.L., Ludwa I.A., Gammage K.L., Mack D.E., Klentrou P. (2010). Relative Importance of Body Composition, Osteoporosis-Related Behaviors, and Parental Income on Bone Speed of Sound in Adolescent Females. Osteoporos. Int..

[26] Klentrou P., Ludwa I.A. (2011). Quantitative Bone Ultrasound Measurements in Young Females 14–23 Years of Age. J. Women’s Health.

[27] Yamakita M., Ando D., Akiyama Y., Sato M., Suzuki K., Yamagata Z. (2019). Association of Objectively Measured Physical Activity and Sedentary Behavior with Bone Stiffness in Peripubertal Children. J. Bone Miner. Metab..

[28] Vlachopoulos D., Barker A.R., Ubago-Guisado E., Williams C.A., Gracia-Marco L. (2018). The Effect of a High-Impact Jumping Intervention on Bone Mass, Bone Stiffness and Fitness Parameters in Adolescent Athletes. Arch. Osteoporos..

[29] Petit M.A., Beck T.J., Kontulainen S.A. (2005). Examining the Developing Bone: What Do We Measure and How Do We Do It?. J. Musculoskelet. Neuronal. Interact..

[30] Gracia-Marco L., Vicente-Rodríguez G., Casajús J.A., Molnar D., Castillo M.J., Moreno L.A. (2011). Effect of Fitness and Physical Activity on Bone Mass in Adolescents: The HELENA Study. Eur. J. Appl. Physiol..

[31] Saint-Maurice P.F., Laurson K., Welk G.J., Eisenmann J., Gracia-Marco L., Artero E.G., Ortega F., Ruiz J.R., Moreno L.A., Vicente-Rodriguez G. (2018). Grip Strength Cutpoints for Youth Based on a Clinically Relevant Bone Health Outcome. Arch. Osteoporos..

[32] Ludwa I.A., Falk B., Ward W.E., Gammage K.L., Klentrou P. (2017). Mechanical, Biochemical, and Dietary Determinants of the Functional Model of Bone Development of the Radius in Children and Adolescents. Appl. Physiol. Nutr. Metab..

[33] Riddoch C.J., Mattocks C., Deere K., Saunders J., Kirkby J., Tilling K., Leary S.D., Blair S.N., Ness A.R. (2007). Objective Measurement of Levels and Patterns of Physical Activity. Arch. Dis. Child..

[34] Yao M., Ludwa I., Corbett L., Klentrou P., Bonsu P., Gammage K., Falk B. (2011). Bone Speed of Sound and Physical Activity Levels of Overweight and Normal-Weight Girls and Adolescents. Pediatr. Exerc. Sci..

[35] Lorbergs A.L., Farthing J.P., Baxter-Jones A.D.G., Kontulainen S.A. (2011). Forearm Muscle Size, Strength, Force, and Power in Relation to PQCT-Derived Bone Strength at the Radius in Adults. Appl. Physiol. Nutr. Metab..

[36] Blimkie C., Gisolfi C.V., Lamb D.R. (1989). Age- and Sex-Associated Variation in Strength During Childhood: Anthropometric, Morphologic, Neurologic, Biomechanical, Endocrinologic, Genetic and Physical Activity Correlates. Perspective in Exercise Science and Sports Medicine.

[37] Clerke A.M., Clerke J.P., Adams R.D. (2005). Effects of Hand Shape on Maximal Isometric Grip Strength and Its Reliability in Teenagers. J. Hand Ther..

[38] Gerodimos V. (2012). Reliability of Handgrip Strength Test in Basketball Players. J. Hum. Kinet..

[39] Mirwald R.L., Baxter-Jones A.D.G., Bailey D.A., Beunen G.P. (2002). An Assessment of Maturity from Anthropometric Measurements. Med. Sci. Sports Exerc..

[40] Moore S.A., Moore M., Klentrou P., Sullivan P., Falk B. (2010). Maturity Status in Male Child and Adolescent Athletes. J. Sports Med. Phys. Fitness.

[41] Mentzel H.-J., Vilser C., Eulenstein M., Schwartz T., Vogt S., Böttcher J., Yaniv I., Tsoref L., Kauf E., Kaiser W.A. (2005). Assessment of Skeletal Age at the Wrist in Children with a New Ultrasound Device. Pediatr. Radiol..

[42] Kowalski K.C., Crocker P.R.E., Faulkner R.A. (1997). Validation of the Physical Activity Questionnaire for Older Children. Pediatr. Exerc. Sci..

[43] Janz K.F., Lutuchy E.M., Wenthe P., Levy S.M. (2008). Measuring Activity in Children and Adolescents Using Self-Report: PAQC and PAQ-A. Med. Sci. Sports Exerc..

[44] Godin G., Shephard R.J. (1985). A Simple Method to Assess Exercise Behavior in the Community. Can. J. Appl. Sport. Sci..

[45] Jacobs D.R., Ainsworth B.E., Hartman T.J., Leon A.S. (1993). A Simultaneous Evaluation of 10 Commonly Used Physical Activity Questionnaires. Med. Sci. Sports Exerc..

[46] Sallis J.F., Buono M.J., Roby J.J., Micale F.G., Nelson J.A. (1993). Seven-Day Recall and Other Physical Activity Self-Reports in Children and Adolescents. Med. Sci. Sports Exerc..

[47] Scerpella T.A., Tuladhar P., Kanaley J.A. (2002). Validation of the Godin-Shephard Questionnaire in Prepubertal Girls. Med. Sci. Sports Exerc..

[48] Kaplan D. (2008). Structual Equation Modeling: Foundations and Extensions.

[49] Kline R. (2015). Principles and Practice of Structural Equation Modeling.

[50] Nader P.R. (2008). Moderate-to-Vigorous Physical Activity from Ages 9 to 15 Years. JAMA.

[51] Ivuskans A., Lätt E., Mäestu J., Saar M., Purge P., Maasalu K., Jürimäe T., Jürimäe J. (2013). Bone Mineral Density in 11–13-Year-Old Boys: Relative Importance of the Weight Status and Body Composition Factors. Rheumatol. Int..

[52] Bass S.L., Eser P., Daly R. (2005). The Effect of Exercise and Nutrition on the Mechanostat. J. Musculoskelet. Neuronal. Interact..

[53] Bass S.L., Saxon L., Corral A.-M., Rodda C.P., Strauss B.J.G., Reidpath D., Clarke C. (2005). Near Normalisation of Lumbar Spine Bone Density in Young Women with Osteopenia Recovered from Adolescent Onset Anorexia Nervosa: A Longitudinal Study. J. Pediatr. Endocrinol. Metab..

